# Serum D-dimer is not predictive of placenta-mediated complications in pregnancy at high risk: The multicentric prospective cohort AngioPred study

**DOI:** 10.3389/fcell.2023.1115622

**Published:** 2023-01-12

**Authors:** Agathe Hovine, Céline Chauleur, Christophe Gauld, Florence Rancon, Jean-Christophe Gris, Brigitte Tardy, Antoine Giraud, Tiphaine Raia-Barjat

**Affiliations:** ^1^ Department of Gynecology and Obstetrics, Centre Hospitalier Universitaire de Saint-Étienne, Saint-Étienne, France; ^2^ INSERM U1059 SAINBIOSE, Université Jean Monnet, Saint-Étienne, France; ^3^ Department of Psychiatry, Centre Hospitalier Universitaire de Saint-Étienne, Saint-Étienne, France; ^4^ INSERM, Centre d’Investigation Clinique 1408, Saint-Étienne, France; ^5^ Laboratory of Hematology, Centre Hospitalier Universitaire de Nîmes, et université de Montpellier, Saint-Étienne, France; ^6^ I.M. Sechenov First Moscow State Medical University, Moscow, Russia; ^7^ Institut Desbrest d’Epidémiologie et de Santé Publique UMR INSERM - Université de Montpellier, Montpellier, France; ^8^ Laboratory of Hematology, Centre Hospitalier Universitaire de Saint-Étienne, Saint-Étienne, France; ^9^ Neonatal Intensive Care Unit, Centre Hospitalier Universitaire de Saint-Étienne, Saint-Étienne, France

**Keywords:** preeclampsia, D-dimer, longitudinal study, hypercoagulability, placental dysfunction

## Abstract

**Background:** The theory that D-dimer level might has a predictive or diagnostic role in preeclampsia needs to be explored. Aim of the study was to evaluate the association between serum D-dimer level and the occurrence of placenta-mediated complications (PMC) in a pregnant population at high risk.

**Methods:** A prospective multicenter cohort study including 200 pregnant women was conducted.

**Results:** Serum D-dimer increases throughout pregnancy, with the highest levels at the end of gestation. Serum D-dimer level was similar for women with PMC and with no complication. Serum D-dimer level was not different in women with preeclampsia *versus* uncomplicated women. Serum D-dimer level was not different in women with early or late preeclampsia *versus* uncomplicated women.

**Conclusion:** This result suggests that serum D-dimer level was not predictive of the PMC occurrence. This corroborates the fact that the origin of PMC based more on immunity than in hemostasis.

## Introduction

Placenta-mediated complications (PMC) are a heterogeneous group of multisystemic disorders. These can be maternal (preeclampsia (PE), eclampsia, placental abruption, hemolysis elevated liver enzymes low platelets (HELLP) syndrome) or fetoplacental (intrauterine growth retardation (IUGR), *in utero* fetal death, recurrent spontaneous miscarriages). PMC complicates between 2% and 8% of pregnancies (Duley, 2009). Predicting and preventing these potentially serious complications is a major challenge for patients and their fetuses. The risk factors for PMC are well identified, but there is no validated screening strategy to predict the occurrence of PMC in these high-risk patients (Eskenazi et al., 1991; Duckitt and Harrington, 2005). The pathogenesis of PE is not fully understood but is considered to be multifactorial, centered on endothelial dysfunction and complex interactions between inflammatory and coagulation pathways. It is associated with a fibrin deposit in the micro-vascularization, responsible for lack of placental perfusion, intrauterine growth retardation, and dysfunction of maternal organs (Phipps et al., 2019). Several studies have investigated whether PMC are associated with changes in the hemostatic system (Gris et al., 2019). Previous studies have already looked at the use of angiogenic factor assay to predict PMC (Raia-Barjat et al., 2019).

D-dimer is the smallest fragment of fibrin degradation products. Fibrinolysis is the process of physiological dissolution of blood clots or thrombi made up of fibrin by plasmin. Plasmatic D-dimer is an indirect marker of the activation of coagulation followed by thrombolysis (Olson, 2015). D-dimer level is used for the diagnosis of venous thromboembolic disease (VTE) in the non-pregnant population. D-dimer level is significantly increased during pregnancy and proportional to gestational age (Nishii et al., 2009; Jeremiah et al., 2012; Wang et al., 2013; Hedengran et al., 2016). This makes it a non-specific test during pregnancy (Baboolall et al., 2019). D-dimer values during pregnancy are above normal in 15% of patients in the first trimester, 71% in the second trimester, and 96% in the third trimester (Wang et al., 2013). The use of standard D-dimer thresholds during pregnancy leads to misinterpretation of the results. Recent studies have attempted to establish new reference intervals for the level of D-dimer in pregnant women based on the trimester of pregnancy in order to discuss the value of their analysis (Hansen et al., 2011; Réger et al., 2013; Wang et al., 2013). An increased level of D-dimer in pregnant women with PE was described compared to pregnant women with normal blood pressure (Schjetlein et al., 1997). Since activation of blood coagulation occurs at the early stage of the disease, the increase in D-dimer could therefore occur before the onset of hypertension. Thus, the theory that D-dimer level might has a predictive or diagnostic role in preeclampsia needs to be explored (Portelinha et al., 2009). It seems important to identify sensitive and specific biomarkers to facilitate the screening of patients at risk of occurrence or recurrence of PMC, allowing a more rapid diagnosis and improving monitoring. However, few studies have compared D-dimer for predicting PE and IUGR in a high-risk population.

The main objective of this study was to evaluate the relation between serum D-dimer level and the occurrence of PMC. Secondary objectives were (Duley, 2009) to evaluate the association between serum D-dimer level and the occurrence of PE with or without IUGR and IUGR alone; (Eskenazi et al., 1991); to compare serum D-dimer levels between patients with early PE < 34 weeks, with late PE ≥ 34 weeks and uncomplicated patients.

## Materials and methods

### Study population

Our study is based on data from the AngioPred study, as described (Raia-Barjat et al., 2019). The AngioPred study is a prospective multicenter cohort study conducted between June 2008 and October 2010 in the Obstetrics and Gynecology department of Saint-Étienne and Nîmes University Hospitals and the Laboratory of Hematology in Nimes University Hospital.

Inclusion criteria were: (Duley, 2009): diabetes (in diet or with insulin therapy), Eskenazi et al. (1991) hypertension (previously treated before pregnancy or hypertension >140/90 twice before 20 weeks), Duckitt and Harrington (2005) obesity (Body Mass Index ≥30 kg m^−2^) (Phipps et al., 2019) maternal age older than 38 years, Gris et al. (2019) chronic kidney disease (proteinuria ≥300 mg for 24 h or creatininemia ≥1.5 mg/dl before 20 weeks), Raia-Barjat et al. (2019) systemic lupus erythematosus; Olson (2015) antiphospholipid syndrome; Hedengran et al. (2016) family history of cardiovascular disease or venous thromboembolism (VTE) in first degree relatives (Nishii et al., 2009) biological thrombophilia without any personal history of VTE or PMC, Jeremiah et al. (2012) a history of one or more episodes of PMC or (Wang et al., 2013) personal history of VTE. The exclusion criteria were: (Duley, 2009): twin pregnancies; (Eskenazi et al., 1991); patients with a history of fetal death due to congenital malformations, Rh incompatibility, or infectious cause; (Duckitt and Harrington, 2005); IUGR which etiology was of chromosomal, genetic, or infectious origin; (Phipps et al., 2019); the presence of any PMC or VTE at inclusion.

All patients were included before 20 weeks and gave their written consent. At inclusion, demographic data were collected by interview, physical examination, and consultation of the obstetrical medical record. Blood samples provided in the protocol were taken in complement to the conventional laboratory tests for the monitoring of pregnancy.

### Blood collection

Blood samples were collected at the collection center of the University Hospital of Saint-Étienne and Nîmes at 20, 24, 28, 32, and 36 weeks of gestation, totaling five samples per patient. The samples were immediately sent to laboratories for analysis, then centrifuged, aliquoted, and stored at −80°C. Each analysis was then performed blind to other analyses. All samples from the same patient were grouped in the same series of assays.

### Biological analysis

The assays were carried out by the hematology laboratory of Saint-Étienne University Hospital. The analyzes were carried out after thawing in a water bath at 37°C., 10 min. A sandwich-type enzyme-linked immunosorbent assay (ELISA) with the ASSERACHROM^®^ D-DI kit (Diagnostica STAGO) was carried out for the determination of serum D-dimer level at 20, 24, 28, 32, and 36 weeks.

### Evaluation criteria

The primary outcome was the occurrence or recurrence of any PMC diagnosed according to the following criteria: (Duley, 2009): PE with or without IUGR. PE was defined according to the ISSHP (International Society for the Study of Hypertension in Pregnancy criteria) (Tranquilli et al., 2014). PE was diagnosed if a previously normotensive woman had new-onset hypertension (>140 mmHg systolic or >90 mmHg diastolic) after 20 weeks of gestation associated with proteinuria (spot urine protein/creatinine >30 mg/mmol [0.3 mg/mg] or >300 mg/day or at least 1 g/L [‘2 + ’] on dipstick testing) or other maternal organ dysfunction (renal insufficiency, liver involvement, neurological complications, hematological complications); (Eskenazi et al., 1991); IUGR without PE defined by a birthweight ≤ to the 10th centile (According to the AUDIPOG formula) with umbilical Doppler abnormalities. This formula calculates the exact percentile of birth weight from gestational age at birth, sex, and birth weight (Mamelle et al., 1996).

The secondary outcomes were: (Duley, 2009): the occurrence or recurrence of a PE with our without IUGR as defined just before; (Eskenazi et al., 1991); the occurrence of early PE (<34 weeks) and late PE (≥34 weeks).

### Statistical analysis

Statistical analyses were performed using XlSTAT^®^. Qualitative data were presented as absolute and relative frequencies (expressed in %). The qualitative variables were compared by the Chi-square test or by Fisher’s exact test if the numbers were insufficient. Quantitative variables were described by mean and standard deviation, and median and interquartile range and were compared by Student’s *t*-test. In the case of a non-normally distributed variable (assessed by Shapiro−Wilk test), a Wilcoxon-Mann−Whitney test was performed. For comparison of D-dimer level at each gestational age and for comparison between patients with PE, with IUGR, and uncomplicated patients, group differences were assessed using the Kruskal-Wallis test. As distribution was skewed, a Dunn *post hoc* test was assessed for differences between the two groups. Results were reported as boxplots. All hypothesis tests were performed at the 0.05 significance level, so *p* < 0.05 was considered significant.

### Ethics

The Ethics Committee and Institutional Review Board of the University Hospital of Saint-Étienne approved the protocol in March 2008. The study is registered with the ClinicalTrials.gov (identifier NCT00695942). The clinical investigation was performed according to the Helsinki Declaration of 1975, as revised in 1996. All women had given their informed consent to participate.

## Results

### Clinical characteristics

**FIGURE 1:**
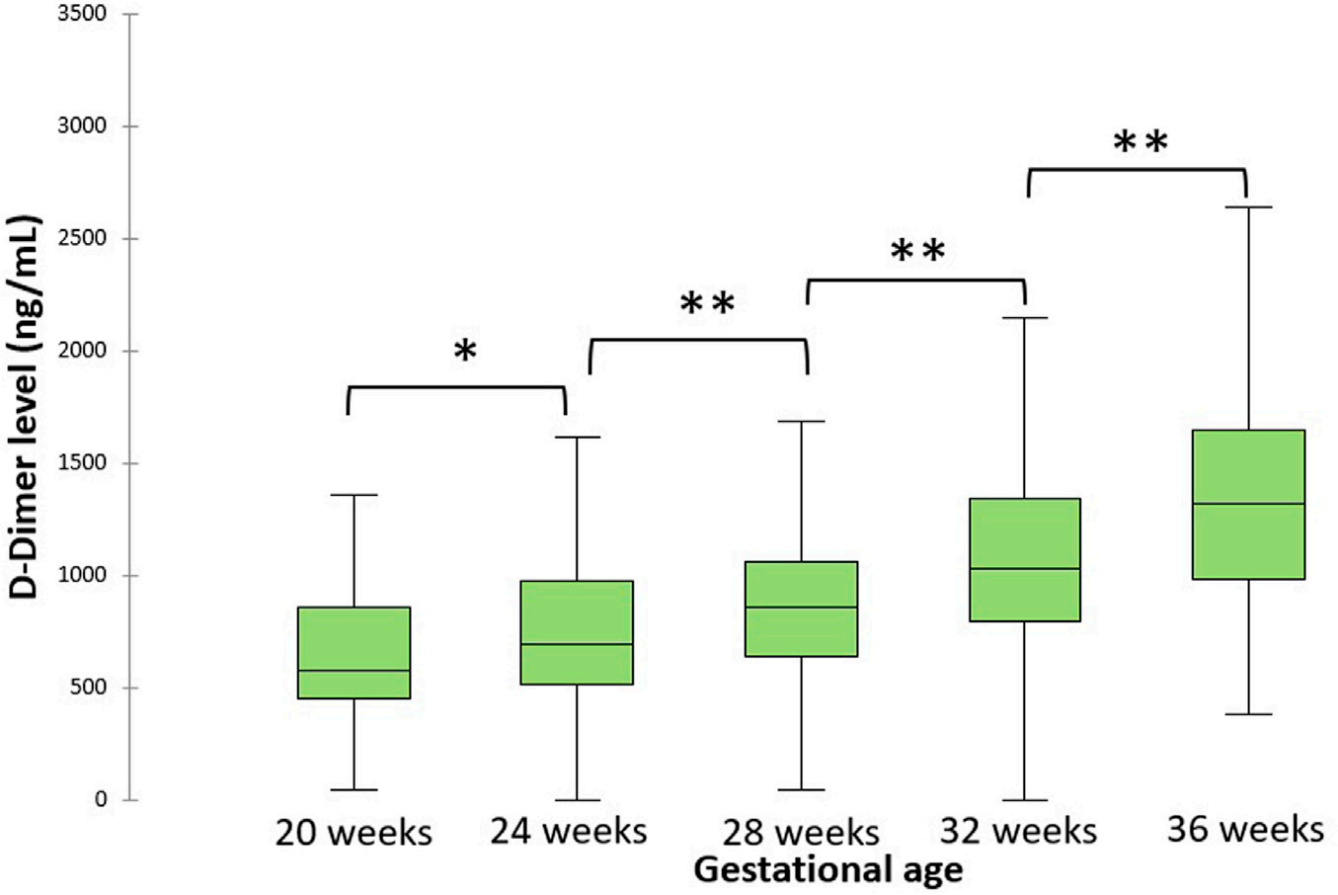
Evolution of D-dimer levels during pregnancy for all population. The central horizontal bars are the medians. The lower and upper limits of the boxes are the first and third quartiles. *p-value < .05; **p-value < .001.

Between June 2008 and October 2010, 200 pregnant women were included in the study. Demographic data and inclusion criteria are summarized in Table 1. History of PMC concerned 69.95% of the study population. During the study, 45 patients had a PMC. PE occurred in 24 patients, including 9 with IUGR. Nine patients presented an early PE < 34 weeks and 15 a late PE ≥ 34 weeks. Conversely, there was a significant difference between the two groups for chronic hypertension and personal history of PVP (*p* < 0.05; Table 1). The patients with PMC experienced more chronic hypertension and had more PMC history compared to uncomplicated. Other demographic characteristics and inclusion criteria were not different between the groups. For all population, the level of D-dimer increases throughout pregnancy, with the highest levels at the end of gestation (Figure 1).

**TABLE 1:** Patient characteristics at inclusion.

	Total 200 patients	PMC 45 patients	Uncomplicated 155 patients	*p*-Value
Maternal characteristics				
Age, years, mean (SD)	31.8 (5.0)	31.4 (5.1)	31.9 (5.0)	0.62
Gestity, mean (SD)	2.9 (1.9)	2.9 (2.0)	2.9 (1.9)	0.80
Parity, mean (SD)	1.2 (0.9)	1.3 (0.8)	1.2 (1.0)	0.59
BMI, kg/m^2^, mean (SD)	25.4 (6.3)	26.1 (6.3)	25.2 (6.3)	0.26
BMI>30 (kg/m^2^)	37 (19.4)	9 (21.4)	28 (18.8)	0.70
Smoking, n (%)	26 (13.4)	7 (15.6)	19 (12.8)	0.63
Diabetes, n (%)	8 (4.1)	3 (6.7)	5 (3.3)	0.32
Kidney disease, n (%)	5 (2.6)	2 (4.4)	3 (2.0)	0.36
Hypertension, n (%)	18 (9.2)	10 (22.2)	8 (5.3)	0.001
Lupus, n (%)	15 (7.7)	2 (4.4)	13 (8.6)	0.36
Antiphospholipid Syndrome, n (%)	6 (3.1)	1 (2.2)	5 (3.3)	0.70
Personal history of VTE, n (%)	38 (19.3)	7 (15.6)	31 (20.4)	0.47
Personal history of PMC, n (%)	135 (68.9)	38 (84.4)	97 (64.2)	0.01
Familial history of cardiovascular disease or VTE, n (%)	39 (19.9)	6 (13.3)	33 (21.9)	0.21

Categorical variables reported as frequency (percentage) and continuous variables reported as mean ± standard deviation. Abbreviations: BMI, body mass index; VTE, venous thromboembolism; PMC, Placenta-mediated complication.

### Relationship between D-dimer levels and the occurrence of placenta-mediated complications

**FIGURE 2:**
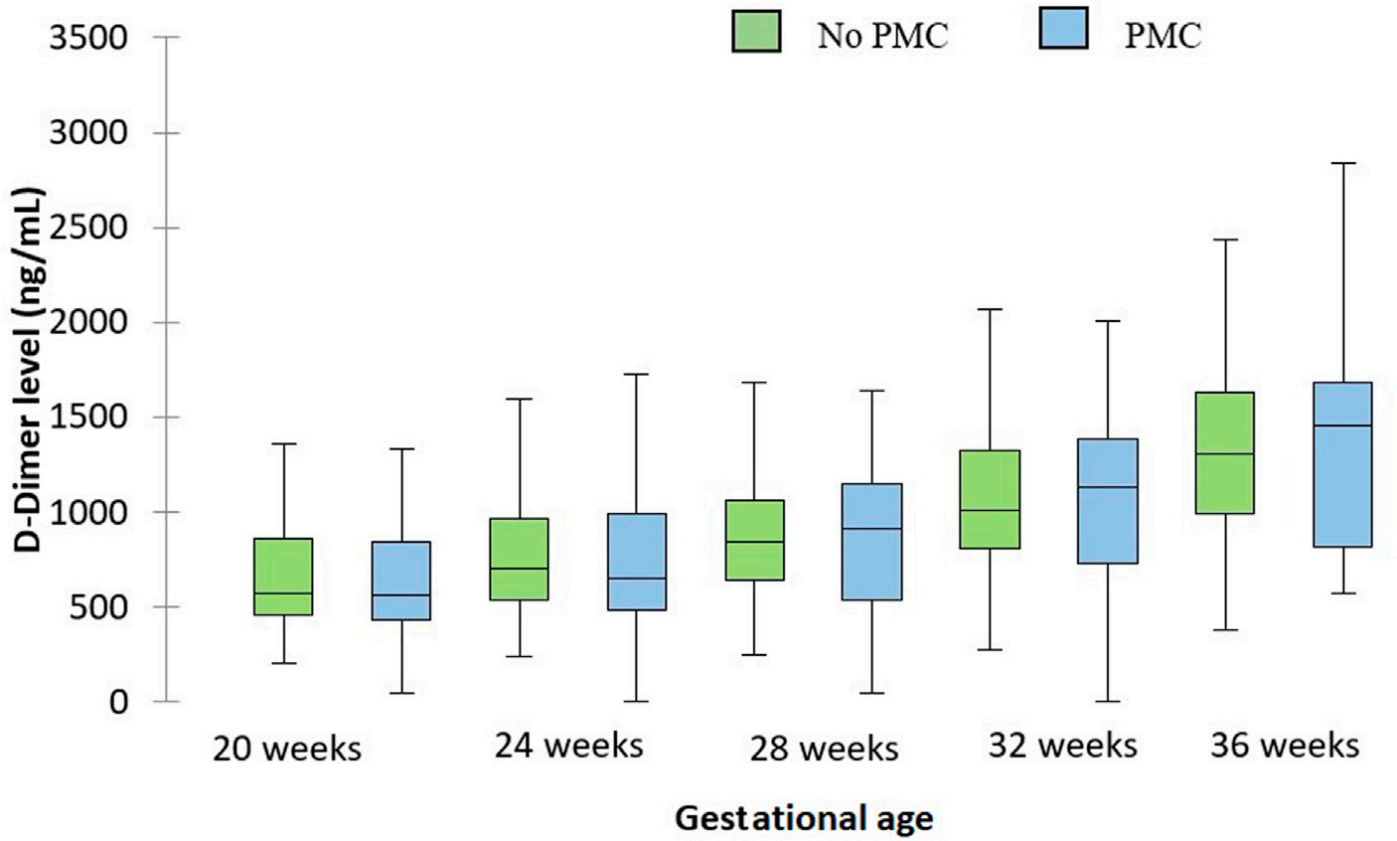
Evolution of D-dimer levels during pregnancy depending on the occurrence of PMC. The central horizontal bars are the medians. The lower and upper limits of the boxes are the first and third quartiles. PMC: placenta-mediated complication.

D-dimer levels were not different between uncomplicated *versus* PMC patients at each gestational age. Results are reported in Figure 2.

### Relationship between D-dimer levels and the occurrence of preeclampsia and IUGR

**FIGURE 3:**
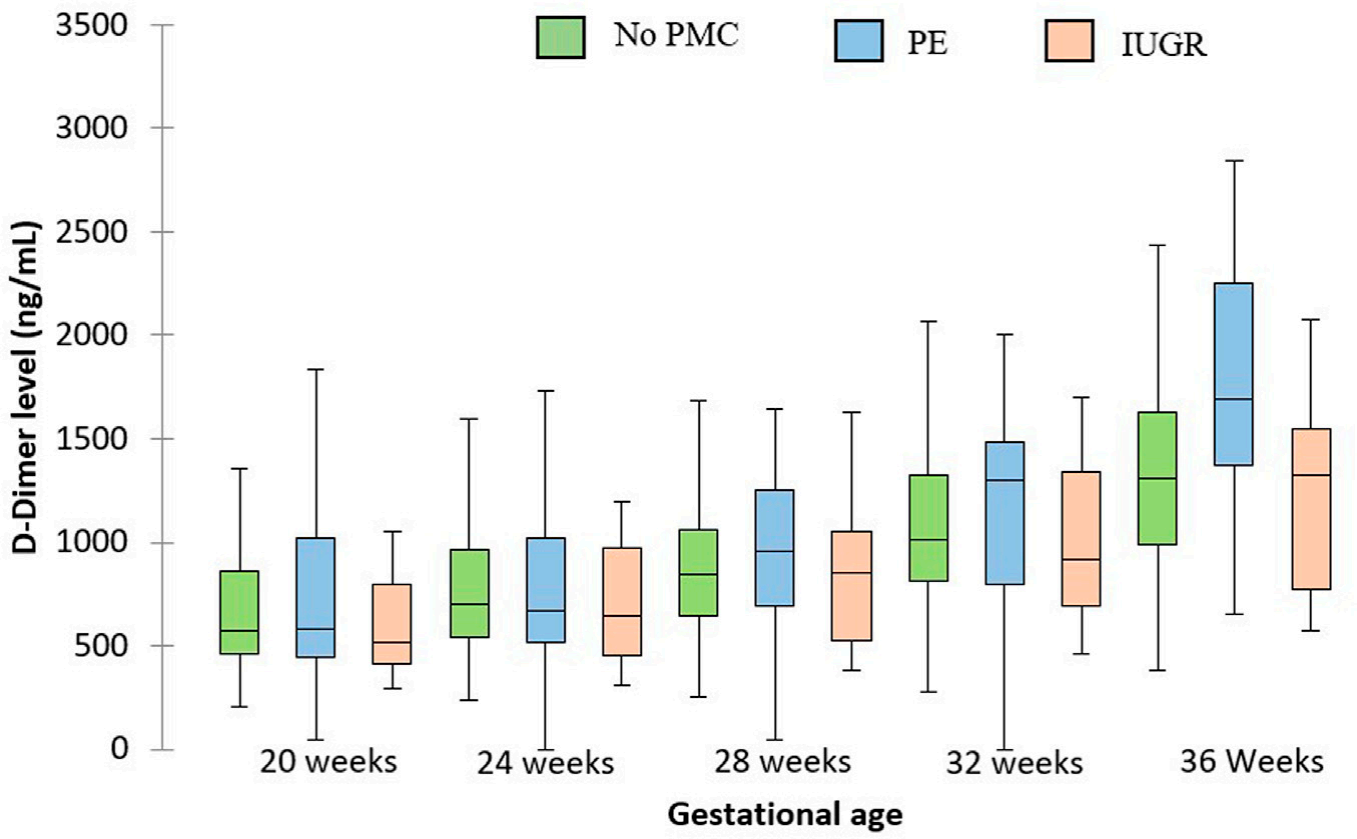
D-dimer levels at each gestational age for patients with PE, IUGR and uncomplicated patients. The central horizontal bars are the medians. The lower and upper limits of the boxes are the first and third quartiles. PMC: placenta-mediated complication.

D-dimer levels were not different in patients with preeclampsia *versus* uncomplicated patients at 28, 32 and 36 weeks (1146.3 *versus* 957.5 ng/ml *p* = 0.48, 1367 *versus* 1167.9 ng/ml 0.31, and 1952 *versus* 1426.94 ng/ml 0.13 respectively). Patients with IUGR had identical serum D-dimer levels than uncomplicated patients. Results are summarized in Figure 3.

### Relationship between D-dimer levels and the occurrence of early and late placenta-mediated complications

**FIGURE 4:**
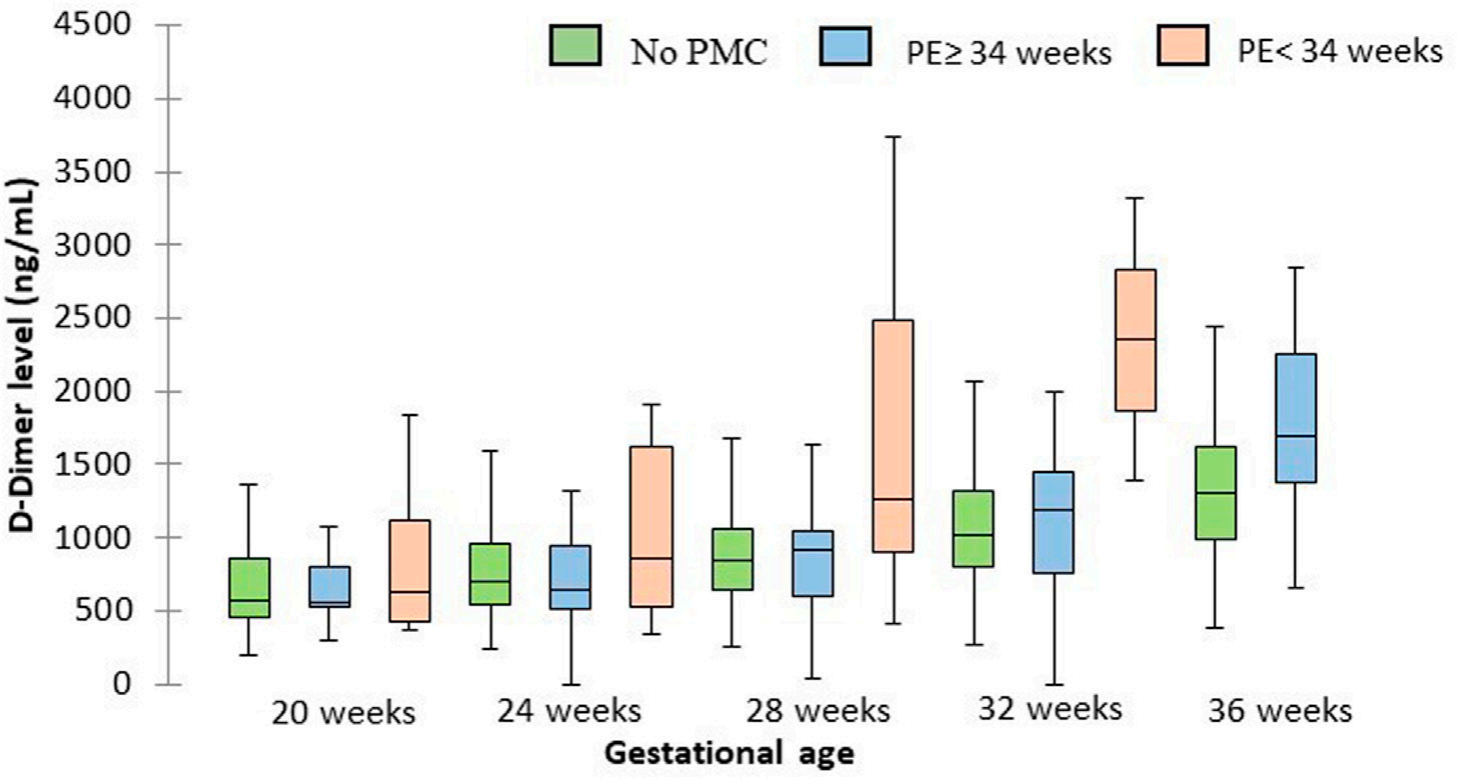
D-dimer levels at each gestational age for patients with early and late PE and for uncomplicated patients. The central horizontal bars are the medians. The lower and upper limits of the boxes are the first and third quartiles. PMC: placenta-mediated complication.

Serum D-dimer levels were not different in patients with early preeclampsia *versus* uncomplicated patients at 28 and 32 weeks (1725.7 *versus* 957.5 ng/ml *p* = 0.28 and 2353.5 *versus* 1167.9 ng/ml *p* = 0.16 respectively). Patients with late preeclampsia had identical serum D-dimer levels than uncomplicated patients at 36 weeks (1952 *versus* 1426.9 ng/ml *p* = .08). Results are summarized in Figure 4.

## Discussion

The level of D-dimer increases throughout pregnancy, with the highest levels at the end of gestation. D-dimer level was not predictive of the PMC occurrence. D-dimer levels could be interesting for the prediction of PE, especially for early PE.

Several authors have studied the evolution of D-dimer levels during pregnancy and in the *postpartum* period and have revealed a gradual increase in the concentration according to gestational age, a conclusion identical to ours. The percentage of patients above the reference threshold of D-dimer (500 ng/ml) for each trimester was 15% in the first, 71% in the second, and 96% of women in the third trimester (Wang et al., 2013).

Three studies with a small number of patients proposed a longitudinal follow-up of D-dimer level throughout pregnancy in a high-risk population. Two studies found no difference in D-dimer level between patients who developed a PE and uncomplicated patients (REF). One study investigated the dosage of D-dimer during pregnancy in patients with a history of PE (Higgins et al., 1998; Hale et al., 2012). One study found an increasing evolution of D-dimer throughout pregnancy, but retained two important periods. Between 12 and 19 weeks, the D-dimer rate was markedly lower in patients in the PE group. Then rapid increase in D-dimer with a peak at 30–34 weeks where the rate of D-dimer was significantly higher in the group of PE patients. We found the same results in the third trimester, but we only assessed D-dimer from 20 weeks and did not have early dosing in the first trimester.

Several authors have studied D-dimer in patients at the time of PE. A meta-analysis in 2012 highlighted a possible diagnostic role for D-dimer level in preeclampsia, particularly in the third trimester of pregnancy (de Barros Pinheiro et al., 2012). On seven studies comparing the rate of D-dimer in PE and normal pregnancies, five studies found a slightly higher rate of D-dimer than in controls, while the other two showed no difference.

D-dimer levels have also been studied to predict the severity of PE. When D-dimer dosage was performed at the time of PE, D-dimer level was increased for patients with severe PE compared to moderate PE (Pinheiro et al., 2014; Baboolall et al., 2019). The dosage of D-dimer appears to be more relevant when PE occurs, with a low predictive value. This result suggests that the D-dimer level is a consequence of PMC and not of a modification preceding its occurrence.

PE is a condition associated with a noticeable exacerbation of hypercoagulation status compared to a normal pregnancy (Hale et al., 2012; Pinheiro et al., 2014). Although the pathogenesis of PE is not fully understood, the activation of inflammatory cytokines and coagulation pathways play a central role. It has been documented that in PE, endothelial dysfunction leads to an increase in tissue plasminogen activator (tPA) and plasminogen activator inhibitor type 1 (PAI-1), with a clear result of hypercoagulability and fibrinolysis (Dusse et al., 2011; Ismail and Higgins, 2011). However, as a manifestation of placental insufficiency, PAI-2, which plays a local role in placental function during pregnancy, is more diminished in severe PE (19,21). Thus, in severe PE, a lower concentration of PAI-2 upregulates the fibrinolytic system, leading to a higher circulating D-dimer level. The high concentration of D-dimer in severe PE is the result of an exaggerated hypercoagulable state and continuous fibrinolysis. This correlates with the concept of the pathophysiology of PE, according to which the formation of microthrombi and excessive deposition of D-dimer affecting several maternal organs as well as the placenta. The result is a placental hypoperfusion and therefore is responsible for complications of PE. Abnormalities in hemostasis appear to be a consequence of PMCs. The origin of PMC based on in genetics factors, pre-existing factors and immunological factors (Phipps et al., 2019).

The strength of the study is the examination of a population of patients at high risk of PMC who were recruited prospectively and followed from 20 weeks to delivery. A limit is to not have a control group of pregnant women at low risk without PMC risk factor.

D-dimer level does not predict the onset of PMC. An increase in the D-dimer level in pregnant women was found in relation to gestational age. Changes in hemostasis appear to occur late in PE onset without knowing whether they are the cause or the consequence of the event. The D-dimer level was more interesting to predict the severity at the time of the onset of PE or IUGR. This corroborates the fact that the origin of PMCs based more on immunity than in hemostasis.

## Data Availability

The raw data supporting the conclusion of this article will be made available by the authors, without undue reservation.
