# Corrigendum: Serum D-dimer is not predictive of placenta-mediated complications in pregnancy at high risk: the multicentric prospective cohort AngioPred study

**DOI:** 10.3389/fcell.2024.1391391

**Published:** 2024-04-04

**Authors:** Agathe Hovine, Céline Chauleur, Christophe Gauld, Florence Rancon, Jean-Christophe Gris, Brigitte Tardy, Antoine Giraud, Tiphaine Raia-Barjat

**Affiliations:** ^1^ Department of Gynecology and Obstetrics, Centre Hospitalier Universitaire de Saint-Étienne, Saint-Étienne, France; ^2^ INSERM U1059 SAINBIOSE, Université Jean Monnet, Saint-Étienne, France; ^3^ Department of Psychiatry, Centre Hospitalier Universitaire de Saint-Étienne, Saint-Étienne, France; ^4^ INSERM, Centre d’Investigation Clinique 1408, Saint-Étienne, France; ^5^ Laboratory of Hematology, Centre Hospitalier Universitaire de Nîmes, et Université de Montpellier, Saint-Étienne, France; ^6^ I.M. Sechenov First Moscow State Medical University, Moscow, Russia; ^7^ Institut Desbrest d’Epidémiologie et de Santé Publique UMR INSERM - Université de Montpellier, Montpellier, France; ^8^ Laboratory of Hematology, Centre Hospitalier Universitaire de Saint-Étienne, Saint-Étienne, France; ^9^ Neonatal Intensive Care Unit, Centre Hospitalier Universitaire de Saint-Étienne, Saint-Étienne, France

**Keywords:** preeclampsia, D-dimer, longitudinal study, hypercoagulability, placental dysfunction

In the published article, an author name was incorrectly written as Christophe Gault. The correct spelling is Christophe Gauld.

The authors apologize for this error and state that this does not change the scientific conclusions of the article in any way. The original article has been updated.

